# Solitary Fibrous Tumor of the Stomach: Diagnostic Pitfalls and Clinical Implications

**DOI:** 10.70352/scrj.cr.25-0314

**Published:** 2025-09-04

**Authors:** Tadakazu Ao, Eiji Shinto, Tenma Ichisawa, Koki Ichio, Takafumi Suzuki, Shohei Mori, Hiroki Abe, Tomomi Koiwai, Makoto Nishikawa, Kiyoshi Nishiyama, Kimi Kato, Hiroaki Takeo

**Affiliations:** 1 Department of Surgery, Self-Defense Forces Central Hospital, Tokyo, Japan; 2 Department of Pathology, Self-Defense Forces Central Hospital, Tokyo, Japan

**Keywords:** solitary fibrous tumor (SFT), stomach, gastrointestinal stromal tumor (GIST), endoscopic ultrasound-guided fine-needle aspiration (EUS-FNA), laparoscopy and endoscopy cooperative surgery (LECS), signal transducer and activator of transcription 6 (STAT6), misdiagnosis

## Abstract

**INTRODUCTION:**

Solitary fibrous tumor (SFT) is a rare mesenchymal neoplasm that most commonly originates in the pleura but can also occur at extrapleural sites, including the abdominal cavity. Among these, primary SFT of the stomach is exceptionally rare. Due to overlapping clinical, endoscopic, and radiologic characteristics, distinguishing SFT from gastrointestinal stromal tumor (GIST) can be particularly challenging. Misdiagnosis may result in inappropriate treatment, such as unnecessary administration of imatinib. Therefore, accurate preoperative differentiation is essential for appropriate management.

**CASE PRESENTATION:**

A 74-year-old man was incidentally found to have a submucosal gastric tumor during a routine health checkup and was referred to our hospital for further evaluation and treatment. Upper gastrointestinal endoscopy revealed a 30-mm subepithelial lesion on the greater curvature of the gastric fundus. Endoscopic ultrasound-guided fine-needle aspiration (EUS-FNA) demonstrated isolated and clustered cells with ovoid to spindle-shaped nuclei. Although not definitive, the combined endoscopic and cytological findings led to a preoperative diagnosis of suspected GIST, and laparoscopy and endoscopy cooperative surgery (LECS) was subsequently performed for local resection. Histopathological examination of the resected specimen revealed an irregular proliferation of spindle cells and nuclear immunopositivity for signal transducer and activator of transcription 6 (STAT6), leading to a final diagnosis of SFT of the stomach. According to Demicco’s risk stratification model, the tumor was classified as low risk. The patient underwent complete resection via LECS and has remained free of recurrence for more than 2.5 years postoperatively.

**CONCLUSIONS:**

This case highlights the difficulty in differentiating SFT from GIST preoperatively and underscores the importance of obtaining sufficient tissue samples to allow for immunohistochemical analysis, particularly STAT6 staining. Recognizing gastric SFT as part of the differential diagnosis is critical to avoid misdiagnosis and ensure appropriate therapeutic decision-making.

## Abbreviations

BMI
: body mass index
EUS-FNA
: endoscopic ultrasound-guided fine-needle aspiration
GIST
: gastrointestinal stromal tumor
LECS
: laparoscopy and endoscopy cooperative surgery
*NAB2*–*STAT6*
: NAB2–STAT6 fusion gene
N/C
: nuclear-to-cytoplasmic (ratio)
SFT
: solitary fibrous tumor
SMA
: smooth muscle actin
STAT6
: signal transducer and activator of transcription 6
TKI
: tyrosine kinase inhibitor
VEGF
: vascular endothelial growth factor
WHO
: World Health Organization

## INTRODUCTION

SFT is a relatively rare mesenchymal neoplasm that most commonly originates in the pleura; however, it can occur at various extrathoracic sites. Although extrapleural SFTs have become increasingly recognized, primary involvement of the gastrointestinal tract—particularly the stomach—remains exceptionally rare. In this report, we describe a rare case of primary SFT of the stomach that was initially misdiagnosed as a GIST based on endoscopic and cytological findings. This case highlights the diagnostic challenges associated with this entity and is accompanied by a brief review of the literature to underscore key clinical considerations, particularly the serious consequences of pretreatment misdiagnosis.

## CASE PRESENTATION

A 74-year-old man was referred to our hospital for evaluation of a submucosal gastric tumor incidentally detected during a routine health checkup. His medical history was notable for diabetes mellitus. He had no known allergies or significant family history. At the time of presentation, he was asymptomatic. His height was 172 cm, weight 60 kg, and BMI was 20.3. Physical examination revealed no abdominal tenderness or palpable masses. Laboratory investigations, including tumor markers, showed no significant abnormalities.

**Fig. 1:**
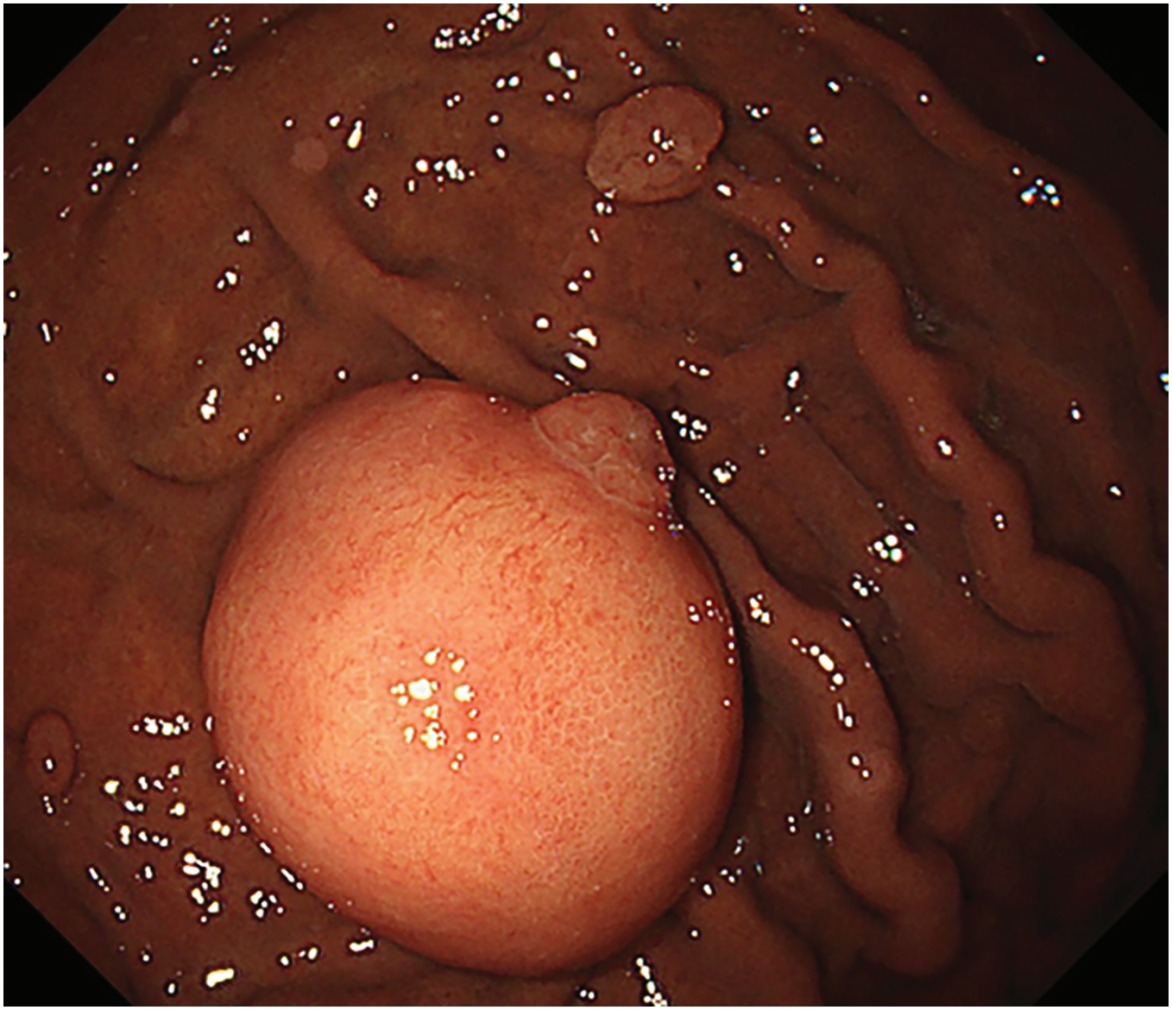
Upper gastrointestinal endoscopic findings. A 30-mm, subpedunculated submucosal tumor was observed on the greater curvature of the gastric fundus during routine endoscopy. The lesion showed a smooth surface and tense appearance without ulceration or bleeding. These non-specific features made preoperative differentiation from gastrointestinal stromal tumor (GIST) difficult.

**Fig. 2:**
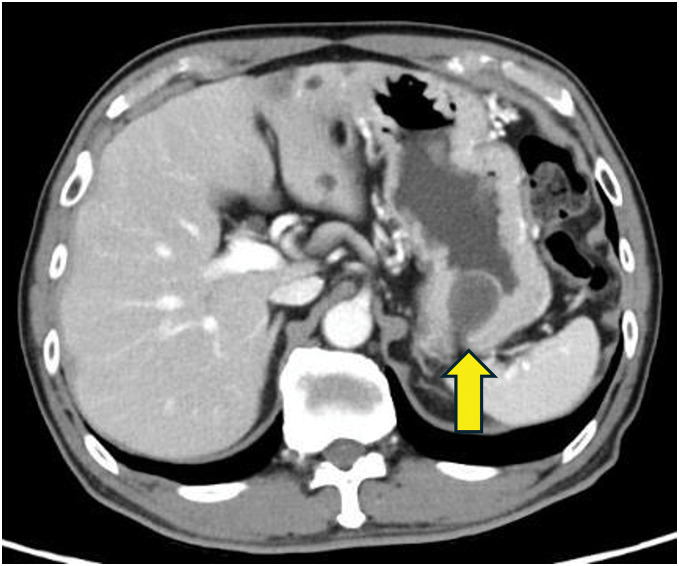
Contrast-enhanced computed tomography findings. Contrast-enhanced CT revealed a 28-mm ovoid submucosal tumor with mild heterogeneous enhancement in the posterior wall of the gastric body (arrow). No evidence of lymphadenopathy or distant metastasis was observed. The tumor was well-circumscribed and lacked invasive features, radiologically resembling a gastrointestinal stromal tumor (GIST).

Abdominal ultrasonography demonstrated a well-circumscribed, hypoechoic mass measuring 26 mm in the fundus of the stomach. Upper gastrointestinal series and endoscopy revealed a 30-mm, smooth-surfaced, subepithelial lesion located on the greater curvature of the gastric fundus (**Fig. 1**). EUS-FNA showed isolated and clustered cells with ovoid or spindle-shaped nuclei. While these findings were insufficient for a definitive diagnosis, the possibility of a neoplastic lesion, including GIST, remained. Contrast-enhanced CT demonstrated a 28-mm submucosal mass with mild heterogeneous enhancement on the posterior wall of the gastric body (**Fig. 2**), without evidence of lymphadenopathy or distant metastasis.

**Fig. 3:**
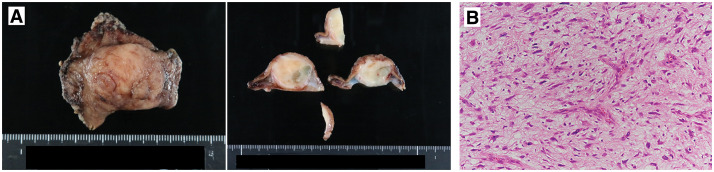
Macroscopic and microscopic findings of the surgically resected specimen. (**A**) Grossly, a submucosal tumor measuring 26 × 24 × 21 mm was located in the central portion of the resected specimen. The cut surface was solid, well-demarcated, and grayish-white in color. (**B**) Histological examination revealed irregular fascicular proliferation of spindle-shaped and stellate tumor cells with pleomorphic, hyperchromatic nuclei. These findings were suggestive of a spindle cell tumor.

Based on these findings, GIST was the primary diagnostic consideration, and LECS was subsequently performed. The patient underwent classical LECS, with an operation time of 280 minutes and estimated blood loss of 50 mL. Gross examination of the resected specimen revealed a well-demarcated submucosal tumor measuring 2.6 × 2.4 × 2.1 cm (**Fig. 3A**). Histologically, spindle to stellate tumor cells with pleomorphic, chromatin-rich nuclei proliferated in irregular fascicles (**Fig. 3B**). Nuclear atypia was observed, including intranuclear inclusions and multinucleated cells.

**Fig. 4:**
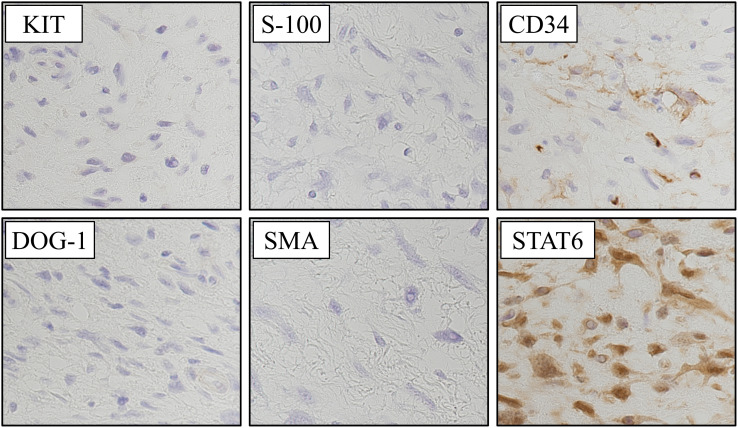
Immunohistochemical staining findings. Tumor cells were immunonegative for KIT (CD117), DOG-1, S-100, and smooth muscle actin (SMA), ruling out gastrointestinal stromal tumor (GIST) and neural tumors. CD34 showed focal positivity. Strong and diffuse nuclear positivity for signal transducer and activator of transcription 6 (STAT6) confirmed the diagnosis of solitary fibrous tumor (SFT).

Immunohistochemical staining was negative for SMA, KIT (CD117), DOG1, and S100; partially positive for CD34; and strongly and diffusely positive for STAT6 in both the nucleus and cytoplasm, confirming the diagnosis of SFT (**Fig. 4**).

According to the risk stratification model proposed by Demicco et al.,^1,2)^ which incorporates age (<55 or ≥55 years), tumor size (<5, 5–<10, 10–<15, or ≥15 cm), mitotic count (per 10 high-power fields: 0, 1–3, or ≥4), and tumor necrosis (<10% or ≥10%) (**Table 1**), only one adverse factor—age—was applicable in this case. The tumor was therefore classified as low risk. The postoperative course was uneventful, and the patient has remained free of recurrence for more than 2.5 years following R0 resection via LECS.

**Table 1:** Risk stratification and clinical outcomes of solitary fibrous tumors proposed by Demicco et al.

A. Risk factors and scoring criteria			
		Three-variable model	Four-variable model
Risk factor		Score	Score
Age	<55	0	0
	≥55	1	1
Tumor size (cm)	<5	0	0
	5 to <10	1	1
	10 to <15	2	2
	≥15	3	3
Mitotic count (/10 high-power fields)	0	0	0
	1–3	1	1
	≥4	2	2
Tumor necrosis	<10%	–	0
	≥10%	–	1
		Total score	Total score
Risk class	Low	0–2	0–3
	Intermediate	3–4	4–5
	High	5–6	6–7

B. Stratified risk classes and long-term prognostic outcomes			
Risk group	Score	N (%)	5 years metastasis-free survival rate
Three-variable model			
Low	0–2	23 (46%)	100%
Intermediate	3–4	17 (34%)	93%
High	5–6	10 (20%)	49%
Four-variable model			
Low	0–3	28 (56%)	100%
Intermediate	4–5	15 (30%)	90%
High	6–7	7 (14%)	27%

## DISCUSSION

SFT is a mesenchymal tumor classified as an intermediate-grade malignancy and categorized as a fibroblastic tumor in the WHO classification of soft tissue tumors.^3)^ Although initially described as a pleural tumor, SFT is now known to occur at various anatomical sites. Among extrapleural cases, the majority arise either in the soft tissues of the extremities or in the abdomen, pelvis, and/or retroperitoneum, with each region accounting for approximately 30%–40% of cases.^3)^ SFTs can occur across a wide age range but are most commonly diagnosed in adults between 40 and 70 years of age, with no clear sex predilection observed.^3)^ In particular, primary SFT of the stomach is extremely rare. A review of the literature identified only 12 previously reported cases. ^4–15)^ The median age at diagnosis among these cases was 68 years (range, 26–85 years), and tumor size ranged from 1 to 16 cm, with a median diameter of 5.4 cm (**Table 2**).

**Table 2:** Summary of case reports

Author	Year	Age	Sex	Tumor size (cm)	Preoperative pathological methods	Preoperative diagnosis	Medical treatment	Positive IHC markers	Negative IHC markers	Follow-up period	Recurrence
Shidam^13)^	1998	77	F	3	FNA (cell block)	SFT	Close follow-up	CD34, Bcl-2, Vimentin, SMA, Collagen IV, Factor XIIIa, HLA DR, CD68	MSA, desmin, pancytokeratin, S-100, CD31, EMA	Not mentioned	Not mentioned
Lee^10)^	2004	70	M	8.5 × 7 × 6	Not performed	Gastric tumor	Wedge resection (open)	CD34, Vimentin	CD117, S-100, Desmin, Pancytokeratin, SMA, CD99	Not mentioned	Not mentioned
Park^12)^	2007	26	M	5.4 × 5.2 × 4	Biopsy	Barium granuloma	Wedge resection	CD34, S-100	CD117, Desmin, SMA	12 months	No
Nabeshima^11)^	2015	43	F	2.7 × 2 × 1.5	Biopsy	Sarcoma	Wedge resection (LECS)	CD34, Bcl-2, MIC-2	CD117, DOG-1, SMA, ALK-1, ALK-EML4	8 months	No
Bosković^6)^	2015	65	F	2.5 × 2.3 × 1	Not mentioned	GIST	Surgical resection	CD34, Vimentin	CD117, DOG-1, S-100, Desmin, SMA, PDGFRA	Not mentioned	Not mentioned
Xiang^15)^	2016	56	M	4.5 × 3	Not mentioned	Not mentioned	Wedge resection (LECS)	CD34, Bcl-2, CD99, Vimentin	CD117, DOG-1, S-100, Desmin, ALK	3 months	No
Inayat^8)^	2017	55	M	7.1 × 6.7	EMR	Submucosal tumor	EMR	CD34, Bcl-2	CD117, DOG-1, S-100, Desmin, ALK	6 months	No
Voth^14)^	2018	68	M	16 × 9	Initial: FNA (cytology) Second: Biopsy	Initial: GIST Second: SFT	Partial gastorectomy	STAT6, CD34	CD117, DOG-1	4 months	Yes (Liver metastasis)
Kimmel^9)^	2019	81	F	7.5	Biopsy (No tumor tissue)	Gastric tumor	Sleeve gastorectomy	STAT6, CD34, Bcl-2, Vimentin, PDGFRA	CD117, DOG-1, S-100, Desmin, SMA, Calponin, ERG, CAM5.2	Not mentioned	Not mentioned
Ababneh^4)^	2020	79	F	6.6	FNA (cell block)	SFT	Not mentioned	STAT6, CD34, Bcl-2	CD117, DOG-1, S-100, Desmin, CD31, ERG-ENDO, AE1/AE3, Synaptophysin	Not mentioned	Not mentioned
Aslam^5)^	2025	85	F	1	EMR	Submucosal tumor	EMR	STAT6, CD34	CD117, DOG1, S100, Desmin, SMA, SOX10	18 months	No
Dong^7)^	2025	67	M	3.1 × 1.7	Not performed	GIST	Laparoscopic gastrectomy	STAT6, CD34, Bcl-2, CD99, Vimentin	CD117, DOG1, S100, CK, SMA, β-catenin	18 months	No
Ao	2025	74	M	2.6 × 2.4	FNA (cytology)	GIST	Wedge resection (LECS)	STAT6, CD34	CD117, DOG-1, S-100, SMA	30 months	No

EMR, endoscopic mucosal resection; F, female; FNA, fine needle aspiration; GIST, gastrointestinal stromal tumor; IHC, immunohistochemical; LECS, laparoscopy and endoscopy cooperative surgery; M, male; MSA, muscle specific actin; SFT, solitary fibrous tumor; SMA, smooth muscle actin; STAT6, signal transducer and activator of transcription 6

There are no specific serum biomarkers for SFT, and the diagnosis is typically based on a combination of imaging and histopathological findings. On contrast-enhanced CT, SFTs often appear as well-circumscribed masses with enhancement equal to or greater than that of the surrounding muscle tissue.^16)^ MRI generally shows low to isointense signals on T1-weighted images and heterogeneous signals on T2-weighted images, with post-contrast images revealing variable enhancement.^17)^ However, these findings are non-specific, and histological examination is essential for a definitive diagnosis.

Histologically, SFT is characterized by a prominent, branching, thin-walled, dilated (staghorn) vasculature.^3)^ The tumor cells are typically short and spindle-shaped, exhibiting a high N/C ratio and relatively scant cytoplasm.^3)^ In 2013, the *NAB2–STAT6* fusion gene was identified in the majority of SFT cases, providing a pivotal molecular hallmark for this tumor entity.^18,19)^ Subsequently, the 2020 revision of the WHO classification of soft tissue tumors incorporated the presence of this fusion gene as a defining diagnostic criterion for SFT. As a surrogate marker, nuclear expression of STAT6 detected by immunohistochemistry has emerged as a highly sensitive and specific tool for diagnosis.^3)^ Most SFTs demonstrate strong and diffuse nuclear positivity for STAT6, making this marker indispensable for distinguishing SFT from its histologic mimics.^20–22)^

In the present case, EUS-FNA was performed preoperatively; however, only conventional cytological smears were prepared without cell block specimens, and immunohistochemical analysis was not performed. While immunostaining generally requires formalin-fixed, paraffin-embedded cell block specimens, EUS-FNA material is generally unsuitable for this diagnostic process due to insufficient cellularity. In this context, the diagnosis of SFT was confirmed postoperatively based on histopathological examination of the resected specimen obtained via LECS. To avoid this diagnostic pitfall, when SFT is included in the differential diagnosis, it is recommended that clinicians consider alternative sampling methods—such as deep biopsy—to obtain adequate tissue for definitive pathological and immunohistochemical evaluation.

The mainstay of treatment for SFT is complete surgical resection with negative margins. Incomplete resection is associated with an increased risk of local recurrence. Lymph node metastasis is exceedingly rare, even in pleural SFTs, where nodal involvement has been reported in up to 7% of cases.^23)^ Notably, none of the reported cases of gastric SFT demonstrated lymph node metastasis. These findings suggest that nodal involvement in SFT of the stomach is also uncommon; therefore, routine lymphadenectomy is not considered necessary.

Local recurrence occurs in approximately 10% of cases, and distant metastases develop in 5%–10%.^24)^ In cases of unresectable SFT, chemotherapy is considered a treatment option for the dedifferentiated subtype, whereas VEGF inhibitors and TKIs are regarded as potential therapeutic options for differentiated tumors.^25)^ Among TKIs, imatinib primarily targets the KIT receptor, which is typically not expressed in SFT, and thus its efficacy is generally limited.^26,27)^

Among previously reported cases of SFT of the stomach, there is one instance in which the tumor was initially misdiagnosed as a GIST based on findings from FNA. The patient received preoperative imatinib therapy, which failed to achieve tumor reduction. A subsequent biopsy revealed nuclear STAT6 positivity, confirming the diagnosis of SFT, and the patient ultimately underwent surgical resection.^14)^ In the present case, had the tumor been larger or considered unresectable, a similar misdiagnosis could have led to inappropriate administration of imatinib. As SFTs are typically c-KIT negative and unresponsive to imatinib, misdirected therapy would likely delay appropriate surgical intervention and expose patients to unnecessary drug-related side effects. Therefore, especially when neoadjuvant treatment is being considered, accurate differentiation between SFT and GIST is essential through the acquisition of adequate tissue for immunohistochemical analysis.

## CONCLUSIONS

We presented a rare case of SFT of the stomach, diagnosed by histopathological and STAT6 immunohistochemical analysis following LECS. This case highlights the challenge of differentiating SFT from GIST based on cytological or imaging findings, and underscores the importance of obtaining sufficient tissue for STAT6 immunostaining to ensure an accurate diagnosis. Maintaining a high index of suspicion for SFT in the differential diagnosis of gastric submucosal tumors is essential, as an inaccurate preoperative diagnosis may lead to inappropriate therapeutic decisions.

## DECLARATIONS

### Availability of data and materials

Data sharing is not applicable to this article as no datasets were generated or analyzed during the current study.

## References

[1] Demicco EG, Park MS, Araujo DM, et al. Solitary fibrous tumor: a clinicopathological study of 110 cases and proposed risk assessment model. Mod Pathol 2012; 25: 1298–306. 22575866 10.1038/modpathol.2012.83

[2] Demicco EG, Wagner MJ, Maki RG, et al. Risk assessment in solitary fibrous tumors: validation and refinement of a risk stratification model. Mod Pathol 2017; 30: 1433–42. 28731041 10.1038/modpathol.2017.54

[3] Demicco EG, Fletcher CD, Han A. Solitary fibrous tumor. In: World Health Organization Classification of Tumours Editorial Board, editor. Soft tissue and bone tumours. 5th ed. Lyon: World Health Organization; 2020. p. 104–8.

[4] Ababneh E, Policarpio-Nicolas MLC. Perigastric solitary fibrous tumor (SFT) diagnosed on fine needle aspiration: a case report and review of literature. Diagn Cytopathol 2020; 48: E27–32. 32628336 10.1002/dc.24544

[5] Aslam S, Chandan A, Shah SS. Solitary fibrous tumor of the stomach in a patient with autoimmune atrophic gastritis: a case report. Int J Surg Pathol 2025; 33: 673–5. 39376087 10.1177/10668969241283492

[6] Bosković T, Zivojinov M, Ilić-Sabo J, et al. Rare solitary fibrous tumor of the stomach: a case report. Vojnosanit Pregl 2015; 72: 1035–8. 26731980 10.2298/vsp140131098b

[7] Dong LY, Li YC, Tong HC, et al. Primary solitary fibrous tumor of the stomach: a case report and a review of literature. Medicine (Baltimore) 2025; 104: e41096. 39792758 10.1097/MD.0000000000041096 PMC11730670

[8] Inayat F, Hussain Q, Shafique K, et al. Solitary fibrous tumor of the stomach. ACG Case Rep J 2017; 4: e35. 28286800 10.14309/crj.2017.35 PMC5340651

[9] Kimmel J, Dikman A, Hajdu C. Gastric solitary fibrous tumor causing upper gastrointestinal bleeding. ACG Case Rep J 2019; 6: e00005. 31616714 10.14309/crj.0000000000000005 PMC6657998

[10] Lee WA, Lee MK, Jeen YM, et al. Solitary fibrous tumor arising in gastric serosa. Pathol Int 2004; 54: 436–9. 15144403 10.1111/j.1440-1827.2004.01638.x

[11] Nabeshima K, Tomioku M, Nakamura K, et al. Solitary fibrous tumor of the stomach treated with laparoscopic and endoscopic cooperative surgery. Tokai J Exp Clin Med 2015; 40: 120–3. 26369266

[12] Park SH, Kim MJ, Kwon J, et al. Solitary fibrous tumor arising from stomach: CT findings. Yonsei Med J. 2007; 48: 1056–60. 18159603 10.3349/ymj.2007.48.6.1056 PMC2628185

[13] Shidham VB, Weiss JP, Quinn TJ, et al. Fine needle aspiration cytology of gastric solitary fibrous tumor: a case report. Acta Cytol 1998; 42: 1159–66. 9755675 10.1159/000332106

[14] Voth E, Serio S, Gross J, et al. Solitary fibrous tumor of the stomach with high-grade sarcomatous dedifferentiation. J Surg Case Rep 2018; 2018: rjy307. 30473761 10.1093/jscr/rjy307 PMC6240737

[15] Xiang TG, Liu GX, Li T. An unusual cause of life-threatening upper gastrointestinal bleeding. Gastroenterology 2016; 151: 400–2. 27490228 10.1053/j.gastro.2016.05.003

[16] Dynes MC, White EM, Fry WA, et al. Imaging manifestations of pleural tumors. Radiographics 1992; 12: 1191–201. 1439021 10.1148/radiographics.12.6.1439021

[17] Chun HJ, Byun JY, Jung SE, et al. Benign solitary fibrous tumour of the pre-sacral space: MRI findings. Br J Radiol 1998; 71: 677–9. 9849394 10.1259/bjr.71.846.9849394

[18] Chmielecki J, Crago AM, Rosenberg M, et al. Whole-exome sequencing identifies a recurrent *NAB2-STAT6* fusion in solitary fibrous tumors. Nat Genet 2013; 45: 131–2. 23313954 10.1038/ng.2522 PMC3984043

[19] Robinson DR, Wu YM, Kalyana-Sundaram S, et al. Identification of recurrent *NAB2-STAT6* gene fusions in solitary fibrous tumor by integrative sequencing. Nat Genet 2013; 45: 180–5. 23313952 10.1038/ng.2509 PMC3654808

[20] Doyle LA, Vivero M, Fletcher CD, et al. Nuclear expression of STAT6 distinguishes solitary fibrous tumor from histologic mimics. Mod Pathol 2014; 27: 390–5. 24030747 10.1038/modpathol.2013.164

[21] Schweizer L, Koelsche C, Sahm F, et al. Meningeal hemangiopericytoma and solitary fibrous tumors carry the NAB2-STAT6 fusion and can be diagnosed by nuclear expression of STAT6 protein. Acta Neuropathol 2013; 125: 651–8. 23575898 10.1007/s00401-013-1117-6

[22] Yoshida A, Tsuta K, Ohno M, et al. STAT6 immunohistochemistry is helpful in the diagnosis of solitary fibrous tumors. Am J Surg Pathol 2014; 38: 552–9. 24625420 10.1097/PAS.0000000000000137

[23] Milano MT, Singh DP, Zhang H. Thoracic malignant solitary fibrous tumors: a population-based study of survival. J Thorac Dis 2011; 3: 99–104. 22263072 10.3978/j.issn.2072-1439.2011.01.04 PMC3256508

[24] Han G, Zhang Z, Shen X, et al. Doege-Potter syndrome: a review of the literature including a new case report. Medicine (Baltimore) 2017; 96: e7417. 28682900 10.1097/MD.0000000000007417 PMC5502173

[25] Martin-Broto J, Mondaza-Hernandez JL, Moura DS, et al. A comprehensive review on solitary fibrous tumor: new insights for new horizons. Cancers (Basel) 2021; 13: 2913. 34200924 10.3390/cancers13122913 PMC8230482

[26] Watanabe K, Otsu S, Morinaga R, et al. CD34-negative solitary fibrous tumour resistant to imatinib. BMJ Case Rep 2013; 2013: bcr2013200126. 10.1136/bcr-2013-200126 PMC3736468 23833101

[27] Levard A, Derbel O, Meeus P, et al. Outcome of patients with advanced solitary fibrous tumors: the Centre Léon Bérard experience. BMC Cancer 2013; 13: 109. 23496996 10.1186/1471-2407-13-109 PMC3623626

